# Probing Femtosecond Charge Transfer Dynamics in P3HT–WS_2_ Nanocomposites via Resonant Core Hole Clock Spectroscopy

**DOI:** 10.1021/acsomega.5c06764

**Published:** 2025-10-13

**Authors:** Yunier Garcia-Basabe, Matheus Suenson Cardoso, Jorge Arce-Molina, Dunieskys G. Larrude

**Affiliations:** † 245075 Universidade Federal da Integração Latino-Americana, UNILA, Foz Do Iguaçu 85867-970, Brazil; ‡ School of Engineering, 42524 Mackenzie Presbyterian University, São Paulo 01302-907, Brazil

## Abstract

We investigate the ultrafast charge transfer (CT) dynamics in a nanocomposite of poly­(3-hexylthiophene-2,5-diyl) (P3HT) and tungsten disulfide (WS_2_) using the core-hole clock (CHC) spectroscopy method, complemented by atomic force microscopy (AFM), near-edge X-ray absorption fine structure (NEXAFS), and X-ray photoelectron spectroscopy (XPS). AFM and NEXAFS reveal that the incorporation of WS_2_ modifies the nanoscale morphology and reduces the molecular ordering of P3HT, while XPS evidence the formation of a donor–acceptor interface. CHC analysis demonstrates orbital-specific enhancement of interfacial CT: the transfer time decreases from 8.1 ± 0.5 fs in pure P3HT polymer film to 4.8 ± 0.5 fs in the nanocomposite for π* orbitals and from 2.7 ± 0.5 fs to 1.4 ± 0.5 fs for σ* orbitals. The dependence of τ_CT_ on excitation energy indicates a tunneling-mediated mechanism with enhanced electronic delocalization across the interface. These findings provide direct insight into how WS_2_ incorporation promotes CT at the molecular level, highlighting P3HT–WS_2_ nanocomposites as promising candidates for ultrafast optoelectronic applications.

## Graphical abstract

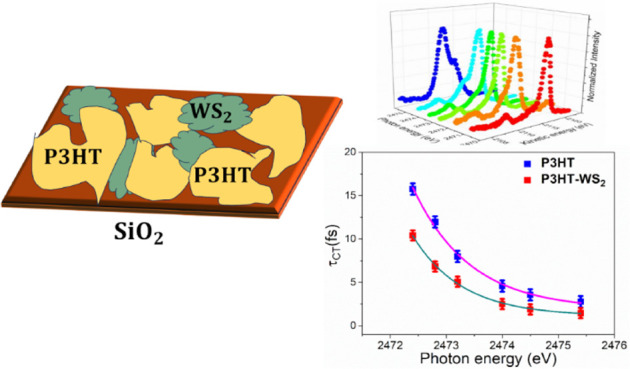

## 1 Introduction

Conjugated semiconducting polymers have emerged as key materials for next-generation flexible and wearable optoelectronic devices, due to their tunable electronic properties, mechanical flexibility, and solution processability.^1–3^ Among these, thiophene-based polymers are particularly attractive due to their high charge-carrier mobility, strong absorption in the visible region, and ease of synthesis and chemical functionalization.^4,5^ Poly­(3-hexylthiophene) (P3HT) is the most widely studied representative in this class and serve as a benchmark material in applications such as organic field-effect transistors (OFETs), organic photovoltaics (OPVs), and photodetectors.^6–8^ However, limitations in long-range charge transport and environmental stability hinder its standalone performance, motivating the development of hybrid materials incorporating inorganic components to improve overall functionality.^9,10^

A promising route to enhance the optoelectronic performance of P3HT involves its integration with two-dimensional (2D) materials. These hybrid systems can overcome intrinsic polymer limitations by enabling broader light absorption, improved charge transport, and increased stability under ambient conditions. Although P3HT exhibits relatively high mobility among conjugated polymers, it still falls short compared to crystalline inorganic semiconductors, particularly regarding long-range transport efficiency and operational stability. The formation of polymer–2D nanocomposites offers synergistic effects such as more efficient charge separation, extended spectral response, and improved durability under ambient conditions.^11,12^ Within the family of 2D materials, transition metal dichalcogenides (TMDs) offer a particularly attractive option due to their tunable band structures, strong light–matter interactions, and potential for favorable interfacial coupling with π-conjugated systems.^13–16^ Tungsten disulfide (WS_2_), in particular, exhibits a direct band gap at the monolayer level, high in-plane charge mobility, and strong optical absorption, making it a compelling candidate for interfacial engineering in hybrid devices.^17–19^ When integrated with WS_2_, P3HT enables efficient charge transfer and energy harvesting mechanisms, making these nanocomposites promising for optoelectronic applications including photodetectors, hybrid solar cells, and field-effect transistors.^20,21^ Several studies have demonstrated the performance enhancements enabled by P3HT–WS_2_ hybrids. Macchia et al.^20^ reported a 10-fold increase in mobility and on/off ratio in electrolyte-gated thin-film transistors based on P3HT–WS_2_ nanotube composites, relative to devices based on pure P3HT. Bruno et al.^21^ showed that incorporating WS_2_ nanotubes into P3HT–CdSe blends leads to fluorescence quenching, consistent with enhanced interfacial electronic interactions in hybrid photovoltaic systems. Kumar et al.^22^ explored WS_2_ nanostructures incorporation into conjugated polymer matrices for light-emitting devices, revealing that WS_2_ nanotubes and nanoparticles functionalized with sodium dodecyl sulfate (SDS) improved electroluminescence and acted as both electron injection and hole blocking layers. Additionally, they observed a blue shift in emission spectra upon deposition of the nanostructures onto P3HT, suggesting changes in the electronic environment of the polymer. Despite these advances, a detailed understanding of interfacial charge transfer mechanism in P3HT–WS_2_ systems remains limited.

The development of high-performance optoelectronic devices based on P3HT–WS_2_ nanocomposites demands a thorough understanding of the charge transfer dynamics at their interface. Key processes such as exciton dissociation, electron tunneling and carrier delocalization, play a decisive role in device performance. While time-resolved techniques like pump–probe spectroscopy,^23^ transient absorption,^24^ and photoluminescence decay^25^ have provided valuable insights into these processes, they often lack element specificity and require complex experimental setups involving ultrafast laser systems and precise synchronization schemes, which limits their applicability in probing site-specific dynamics within heterogeneous nanostructures. In contrast, core-hole clock (CHC) spectroscopy offers an element and orbital-specific probe of electron dynamics by exploiting the ultrafast decay lifetime of core-excited states.^26–28^ By analyzing the intensity ratio between resonant and normal Auger decay channels, CHC allows for the extraction of the electron delocalization times with subfemtosecond to attosecond resolution, enabling the direct investigation of charge transfer across hybrid interfaces.^29,30^

In this work, we investigate the electronic structure and ultrafast charge transfer (CT) dynamics in P3HT–WS_2_ nanocomposites by integrating morphological, spectroscopic, and resonant Auger decay analyses. Atomic force microscopy (AFM) reveals the nanoscale reorganization of P3HT induced by WS_2_ nanosheets, which provides the structural framework for enhanced donor–acceptor interactions. XPS and valence band measurements establish the interfacial electronic alignment, identifying the driving forces behind charge redistribution. Finally, S–KL_2,3_L_2,3_ resonant Auger spectroscopy across the S K-edge probes element- and orbital-specific CT pathways, enabling us to quantify the characteristic time scales of electron delocalization associated with both polymeric and inorganic sulfur states. Together, these complementary results construct a coherent picture linking nanoscale morphology, interfacial electronic structure, and ultrafast charge transfer dynamics, thereby offering new insights into how hybrid organic–inorganic interfaces govern electronic coupling and delocalization processes.

## 2 Experimental Section

### 2.1 Sample Preparations

#### 2.1.1 WS_2_ Exfoliation

Few-layer WS_2_ was prepared using a modified ultrasonic-assisted ball milling exfoliation protocol, as described by Garcia-Basabe et al.^31^ Briefly, 0.5 g of bulk WS_2_ was mixed with NaCl in an agate milling vessel containing 5 mm-diameter agate balls, maintaining a ball-to-powder weight ratio of 7:1. The mixture was processed in a Retsch PM 100 planetary ball mill at 450 rpm for 2 h. After milling, the resulting WS_2_/NaCl powder was rinsed multiple times with deionized water and centrifuged at 4000 rpm for 45 min to remove salt residues. The cleaned material was then dried at 100 °C for 12 h.

Subsequently, 0.15 g of the dry WS_2_ powder was dispersed in 25 mL of *N*-methylpyrrolidone (NMP) in a 50 mL glass vial and subjected to probe sonication (Eco-Sonics/Utronique, 750 W, 20 kHz, 70% amplitude) for 8 h at room temperature. The dispersion was then centrifuged at 4000 rpm for 1 h, and the supernatant containing exfoliated WS_2_ nanosheets was collected for further use. The solvent was then exchanged for isopropyl alcohol (IPA) following the method proposed by Ghasemi et al.,^32^ and the resulting dispersion was dried overnight at 90 °C.

#### 2.1.2 Deposition of P3HT/SiO_2_ Film

The P3HT thin film was prepared following procedures reported in previous studies.^33^ The P3HT polymer was dissolved in chlorobenzene at a concentration of 0.5 mg/mL and deposited onto a precleaned Si/SiO_2_ wafer substrate via spin coating at 1200 rpm for 60 s. To remove residual solvent and enhance polymer crystallinity, the film was placed in an ultrahigh vacuum (UHV) chamber and annealed at 457 K for 2 h. According to previous studies,^34^ polymer films prepared under similar conditions typically have a thickness of approximately 5–10 nm.

#### 2.1.3 Deposition of WS_2_/SiO_2_ Film

The WS_2_ film was deposited onto a Si/SiO_2_ wafer substrate by spin coating a WS_2_ dispersion in (IPA) at 800 rpm for 60 s.

#### 2.1.4 P3HT-WS_2_ Nanocomposite Film

A P3HT-WS_2_ nanocomposite was prepared by mixing 1 mL of a P3HT solution (0.5 mg/mL in chloroform) with 1 mL of a WS_2_ dispersion in chloroform containing 50 wt % of WS_2_. The mixture was stirred for 1 h at room temperature to ensure homogeneity. Subsequently, the dispersion was deposited onto a precleaned SiO_2_/Si wafer via spin coating under conditions similar to those used for the pure P3HT polymer film (1200 rpm for 60 s). The resulting film was then subjected to the same thermal annealing treatment (457 K for 2 h in ultrahigh vacuum).

### 2.2 Sample Characterization

The surface morphology of P3HT/SiO_2_ and P3HT–WS_2_/SiO_2_ thin films was characterized by atomic force microscopy (AFM) using a Bruker Dimension Icon system operated in tapping mode.

X-ray absorption spectroscopy (XAS) and resonant Auger spectroscopy (RAS) measurements were performed at the soft X-ray spectroscopy (SXS) beamline of the Brazilian Synchrotron Light Laboratory (LNLS),^35^ following experimental protocols previously established by our group.^36–38^ A Si(111) double-crystal monochromator with an energy bandwidth of 0.48 eV was used to access the sulfur K-edge region. Near-edge X-ray absorption fine structure (NEXAFS) spectra were acquired in total electron yield (TEY) mode and normalized by the photon flux recorded simultaneously using a gold mesh, in order to correct for beam intensity fluctuations. The incident photon energy was calibrated using the Mo L-edge of a metallic molybdenum standard, with the absorption maximum set to 2520.0 eV. Each spectrum represents the average of at least three scans. Background correction was performed by subtracting a linear pre-edge baseline and applying linear regression fit to the postedge region, using procedures available in the Athena analysis package. The resulting spectra were then normalized to the postedge intensity, where absorption stabilizes, to ensure consistency and minimize the influence of film thickness or other experimental variations.

Sulfur-KL_2,3_L_2,3_ RAS and XPS measurements were conducted in an ultrahigh vacuum (UHV) chamber (base pressure ∼10^–8^ mbar) using a Specs PHOIBOS 150 hemispherical electron energy analyzer. Electrons were collected at a 45° takeoff angle with a pass energy of 20 eV. Binding energy calibration was referenced to the Au 4f_7/2_ core-level at 84.0 eV. The total energy resolution achieved was 0.76 eV. RAS and XPS spectra were fitted using the CasaXPS software package (version 2.3.2) with pseudo-Voigt functions, defined in the Sum Gaussian–Lorentzian (SGL) form as a weighted linear combination of Gaussian and Lorentzian components, where the mixing parameter *m = p/100* varies from GL(0), corresponding to a purely Gaussian line shape, to GL(100), representing a purely Lorentzian profile. In the case of XPS spectra, constraints were imposed during the fitting by fixing the energy separation between the S 2p_3/2_ and S 2p_1/2_ components (≈1.18 eV) with a 2:1 intensity ratio, and between the W 4f_7/2_ and W 4f_5/2_ components (≈2.15 eV) with a 4:3 intensity ratio. The background was modeled using a Shirley-type function. Surface charging effects were monitored by tracking the position of the C 1s (C–C) photoemission peak at 285 eV. Potential radiation-induced degradation of the samples was checked by acquiring NEXAFS spectra after each RAS measurement.

## 3 Results and Discussion

Before discussing the modifications in the electronic structure and charge transfer (CT) dynamics of the P3HT–WS_2_ system, we first examined the film morphology using atomic force microscopy (AFM). Morphological characterization is a critical first step, as nanoscale organization directly influences the electronic coupling between P3HT and WS_2_. Figure 1 presents AFM images of P3HT/SiO_2_ and P3HT–WS_2_/SiO_2_ thin films. The pure P3HT polymer film exhibits globular features typical of polymeric surfaces, with a root-mean-square (rms) roughness of 2.11 nm. Upon incorporation of WS_2_ nanosheets, the composite film develops distinct surface domains and an increased rms roughness of 8.7 nm, indicating changes in P3HT aggregation induced by the 2D material. A similar effect was reported by Nicho et al.^39^ who investigated the morphology of P3HT binary blends with the insulating polymers polystyrene (PS) and poly­(methyl methacrylate) (PMMA). Such heterogeneous domain formation is commonly associated with the creation of donor–acceptor regions that facilitate charge separation and transfer.^40^

**Figure 1:**
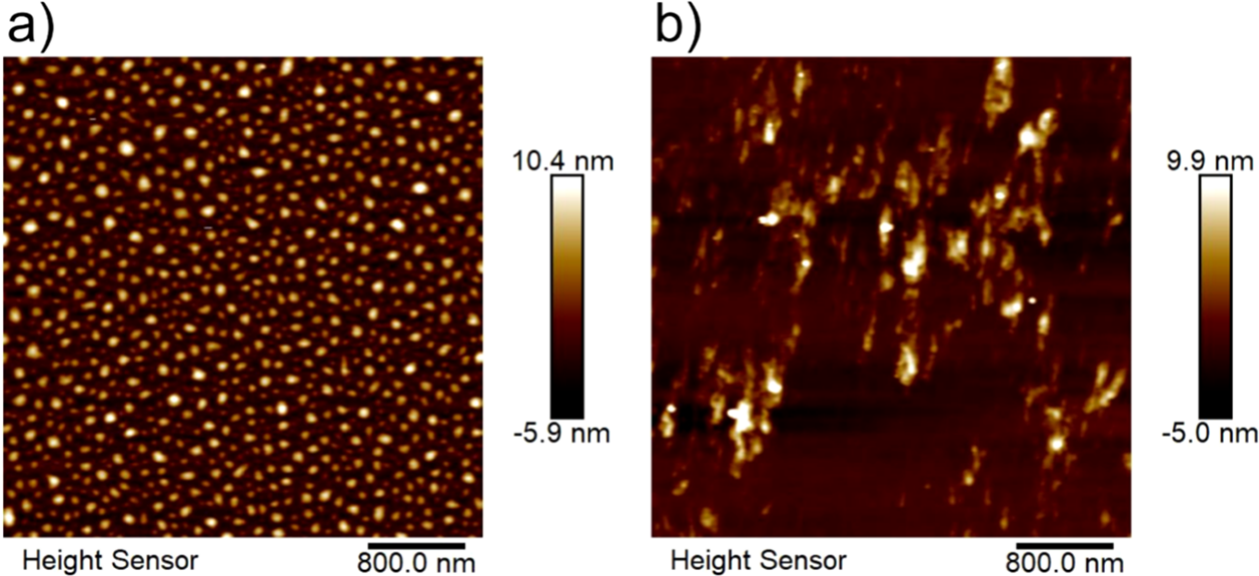
Atomic force microscopy topographic images (4 μm × 4 μm) of (a) P3HT/SiO_2_ and (b) P3HT–WS_2_/SiO_2_ films.

XPS core-level spectra of S 2p and W 4f for P3HT/SiO_2_, WS_2_/SiO_2_, and P3HT–WS_2_/SiO_2_ films are shown in Figure 2a,b. The S 2p spectrum of pure P3HT polymer film (Figure 2a, top) shows the expected spin–orbit doublet peaks at 164.2 eV (S 2p_3_/_2_) and 165.4 eV (S 2p_1_/_2_), corresponding to sulfur atoms in the thiophene rings.^41,42^ In the nanocomposite (Figure 2a, bottom), an additional low-binding energy doublet emerges, indicative of WS_2_-derived sulfur.^43^ The emergence of this low-energy component confirms the successful incorporation of WS_2_ nanosheets into the composite and enables clear chemical differentiation between the organic and inorganic sulfur environments. Additionally, a weak feature at higher binding energy (∼169.5 eV) is detected in the composite, which can be assigned to oxidized sulfur species such as sulfate (SO_4_^2–^), likely originating from surface oxidation.^44^ This oxidation may be driven by interfacial charge transfer between the electron-donating P3HT and electron-accepting WS_2_, which could promote partial oxidation of sulfur at the interface. A reduction in the S 2p peak fwhm from 1.64 eV in the pure P3HT film to 1.13 eV in the P3HT–WS_2_ composite was observed (see Table SI1). We attribute this narrowing to reduced microstructural heterogeneity upon WS_2_ incorporation, consistent with the AFM evidence of domain formation.

**Figure 2:**
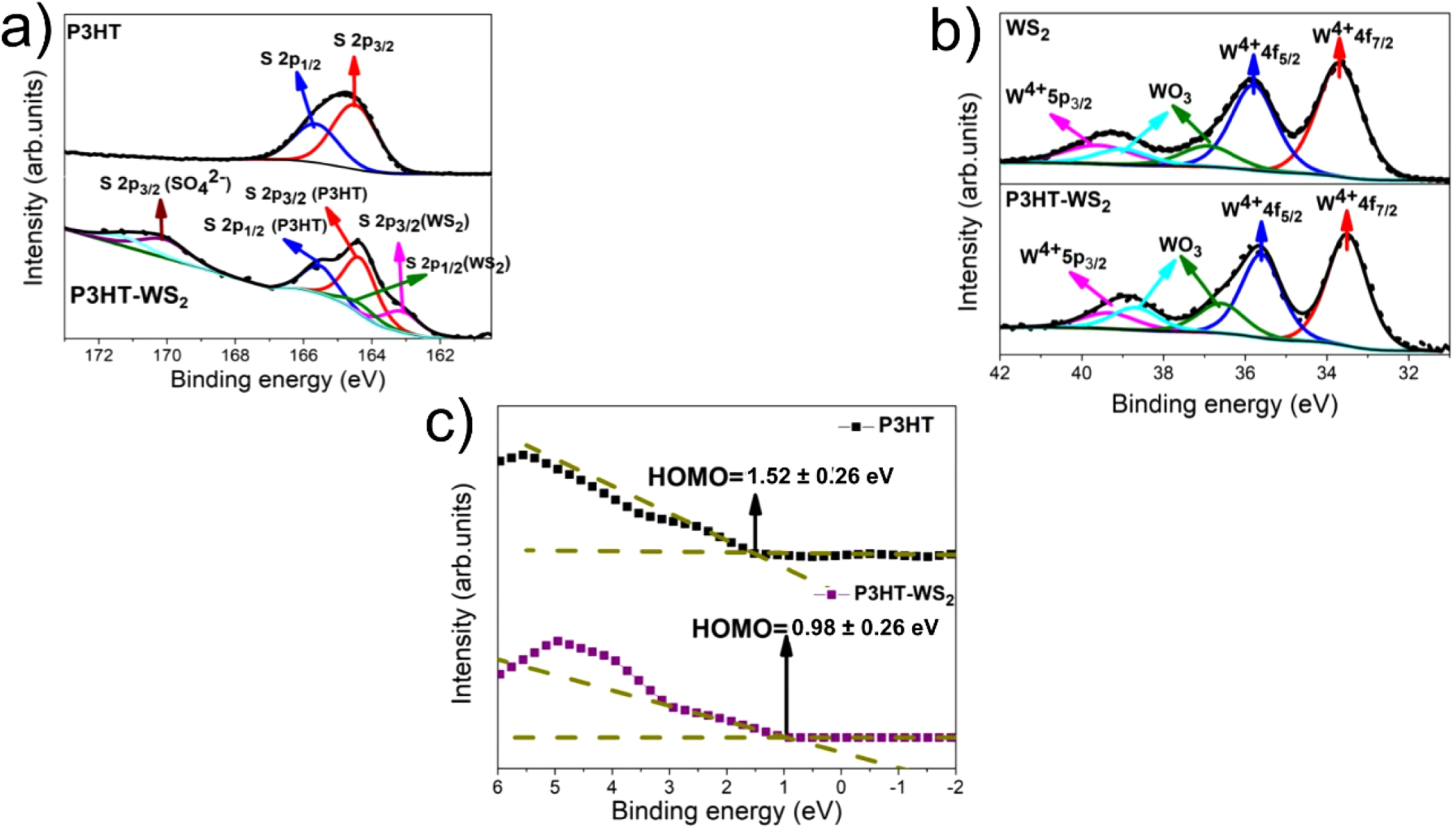
(a) S 2p core-level XPS spectra of P3HT/SiO_2_ and P3HT–WS_2_/SiO_2_ films. (b) W 4f core-level XPS spectra of WS_2_/SiO_2_ and P3HT–WS_2_/SiO_2_ films. (c) Valence-band XPS spectra of P3HT/SiO_2_ and P3HT–WS_2_/SiO_2_ films. All major spectral features are labeled in the figure. XPS measurements were performed using an incident photon energy of 2900 eV.

The W 4f spectra of WS_2_/SiO_2_ and P3HT–WS_2_/SiO_2_ films are shown in Figure 2b. Both spectra exhibit two prominent peaks at 33.7 and 35.8 eV, corresponding to the W^4+^ 4f_7/2_ and 4f_5/2_ components, respectively, features characteristic of the semiconducting 2H phase of WS_2_.^43,45^ Additionally, signals near 36.9 and 39.0 eV are assigned to the W^6+^ 4f_7/2_ and 4f_5/2_ levels, indicating the presence of oxidized tungsten species likely associated with WO_3_ formation.^43^ The W^4+^ 5p_3/2_ component is also discernible in this energy range.^45^ A slight increase in the oxidized W^6+^ species (from 16.1% to 20.5%) and a small reduction in the full width at half-maximum (fwhm) of the W^4+^ 4f peaks (from 1.30 to 1.10 eV) in the P3HT–WS_2_ nanocomposite, relative to pristine WS_2_, may be attributed to interfacial interactions between the conjugated polymer and the WS_2_ nanosheets. Complete fitting parameters are provided in Table SI2.^44,46,47^ The presence of P3HT may facilitate mild surface oxidation of WS_2_, potentially due to residual oxygen exposure or chemical interactions with the polymer matrix. This behavior has been observed in WS_2_ nanotube–polymer systems, where polyelectrolytes (e.g., chitosan) and silane-functionalized composites promote surface oxidation (increased W^6+^) by interfacial chemical interactions.^48,49^ Furthermore, the narrower W^4+^ peaks suggest a more uniform chemical environment around the W atoms, possibly resulting from partial passivation or encapsulation of WS_2_ by the polymer.^50^ Figure 2c presents the valence band XPS spectra of P3HT/SiO_2_ and P3HT–WS_2_ films. The HOMO onset, determined from the low-binding-energy edge of the XPS valence band spectra by extrapolating between the background and a straight baseline, is found at 1.52 ± 0.26 eV below the Fermi level (E_F_, binding energy B.E. = 0 eV) for the pure P3HT film. In the P3HT–WS_2_ nanocomposite, this onset shifts toward lower binding energy, being located at 0.98 ± 0.26 eV relative to E_F_. This shift reflects the formation of a donor–acceptor interface and is consistent with interfacial charge transfer from P3HT to WS_2_.^51^ These findings point to a modified interfacial electronic structure, which may significantly influence the charge transfer dynamics in the nanocomposite system.

Figure 3 shows the sulfur K-edge NEXAFS spectra of WS_2_/SiO_2_, P3HT/SiO_2_, and P3HT–WS_2_/SiO_2_ films. The S K-edge NEXAFS spectrum of the WS_2_/SiO_2_ film (Figure 3a) is characterized by two prominent features below the sulfur ionization threshold (2474.7 eV). The first peak, located at 2470.3 eV, is attributed to transitions from the S 1s core level to unoccupied S 3p_*x*,*y*_ orbitals, while the second feature, around 2472.0 eV, corresponds to transitions into S 3p_*z*_-derived states, as previously reported in the literature.^52,53^ On the other hand, the S K-edge NEXAFS spectrum of the P3HT/SiO_2_ film (Figure 3b) displays two characteristic resonances: the S 1s → π* (S=C) transition at a photon energy of 2472.8 eV, and the S 1s → σ* (S–C) transition, with the transition moment oriented parallel to the polymer backbone, centered at 2474.5 eV.^54,55^ As shown in Figure 3c, the sulfur K-edge NEXAFS spectrum of the P3HT–WS_2_ nanocomposite exhibits features that represent a combination of the characteristic transitions of its individual components. In addition to these expected resonances, the nanocomposite spectrum reveals a pronounced feature at 2482.0 eV, well above the S 1s ionization threshold. While an analogous transition appears in the spectrum of the isolated WS_2_ film, its intensity is notably enhanced in the composite. According to the report by Prouzet et al.,^56^ this transition can be associated with an amount of sulfur atoms in the +6-oxidation state, corresponding to sulfate ions (SO_4_^2–^). This behavior is consistent with the XPS analysis, which reveals an increase in oxidized sulfur species in the nanocomposite, suggesting a modified electronic environment around the sulfur atoms, likely due to interfacial interactions between P3HT and WS_2_.

**Figure 3:**
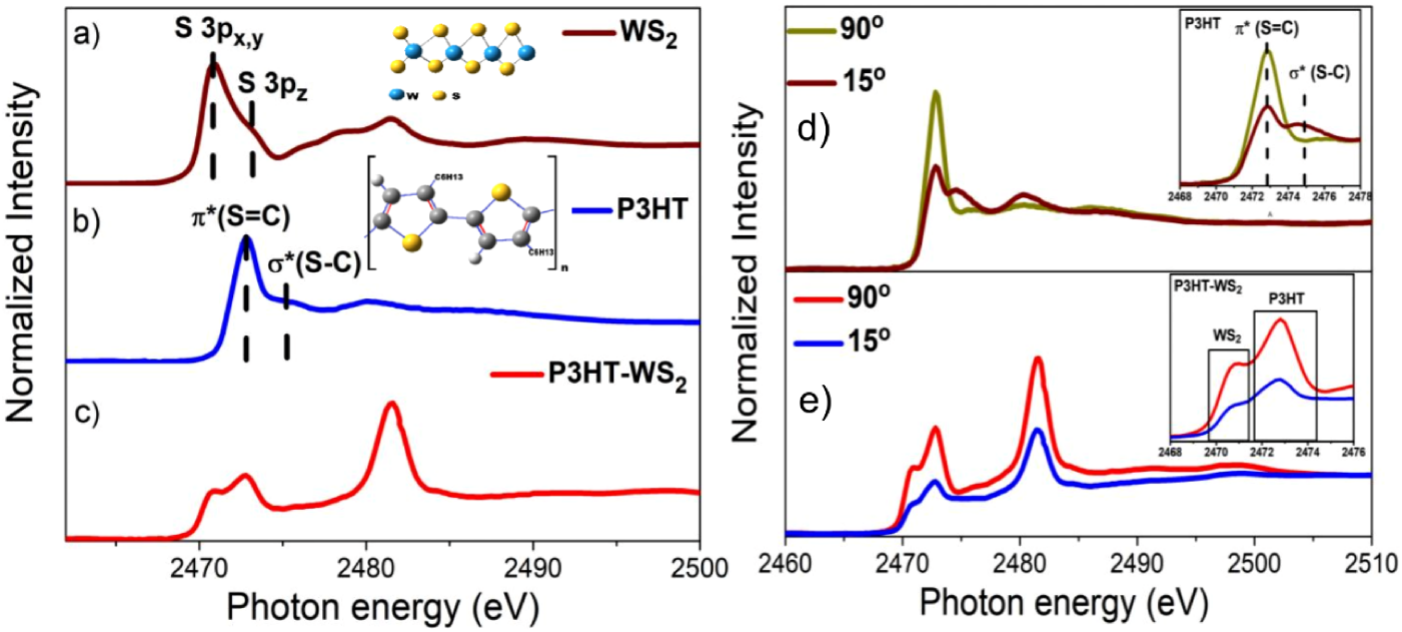
Sulfur K-edge NEXAFS spectra measured at 45° incidence for (a) WS_2_/SiO_2_, (b) P3HT/SiO_2_, and (c) P3HT–WS_2_/SiO_2_ films. Panels d and e show polarization-dependent spectra of P3HT and P3HT–WS_2_, recorded at normal (90°) and grazing (15°) incidence. The insets in d and e highlight the resonance region, while the insets in a and b depict schematic representations of the P3HT molecular structure and the WS_2_ layered structure, respectively.

Figure 3d,e presents angle-resolved S K-edge NEXAFS spectra collected at normal (90°) and grazing (15°) incidence for both pure P3HT polymer film and the P3HT–WS_2_ nanocomposite. Electronic excitations detected in NEXAFS spectra are subject to dipole selection rules and are strongly affected by the directional character of π* and σ* antibonding orbitals in organic systems. Due to this intrinsic anisotropy, variations in absorption intensity with respect to the angle of incidence and polarization of the linearly polarized synchrotron radiation can be exploited to extract information on molecular orientation. Specifically, the transition probability is maximized when the electric field vector of the incoming beam is aligned with the transition dipole moment associated with a particular molecular orbital. For pure P3HT polymer film, clear angular dependence is observed: the intensity of the S 1s → σ* (S–C) transition increases under grazing incidence, while the S 1s → π* (C=S) transition dominates at normal incidence. This dichroism is consistent with a preferential edge-on molecular orientation of the P3HT chains, in which the thiophene rings lie perpendicular to the substrate plane.^38,57^ This molecular orientation is also in agreement with the report by Ikeura-Sekiguchi et al.^54^ for regioregular P3HT, where opposite polarization dependences were also observed, with the σ* transition moment parallel and the π* transition moment perpendicular to the polymer backbone. In contrast, the NEXAFS spectra of the P3HT–WS_2_ nanocomposite show negligible variation with incident angle. Although the spectra measured at grazing incidence exhibit overall lower intensity compared to those acquired at normal incidence, the relative intensities of the π and σ transitions remain essentially unchanged. This reduction in signal is attributed to the limited escape depth of secondary electrons in the TEY mode, which decreases the detection efficiency at grazing incidence angles. This lack of dichroism indicates that the interaction with WS_2_ disrupts the preferential molecular orientation typically observed in neat P3HT films. Such disruption is likely caused by interfacial interactions between P3HT and the WS_2_ surface, which hinder the self-assembly of semicrystalline domains. This observation is consistent with the AFM results, which also reveal morphological changes in P3HT induced by the presence of WS_2_ nanosheets, supporting the notion of disrupted molecular ordering.

To investigate the charge delocalization dynamics in the P3HT–WS_2_ nanocomposite, sulfur KL_2,3_L_2,3_ Auger decay spectra were acquired at various excitation energies across the S K-edge resonance, as shown in Figure 4a. In agreement with previous studies, these Auger spectra consist of ^1^S and^1^D multiplet components as show in Figure 4b.^29,38,55^ For comparison, the resonant Auger spectra of pristine WS_2_ and pure P3HT films are presented in Figure 4c,d, respectively, allowing the individual contributions of each component to be disentangled. The nanocomposite spectra clearly exhibit a coexistence of decay channels from both WS_2_ and P3HT. At lower excitation energies (e.g., 2471.0 eV), the spectral response is dominated by WS_2_, while at higher photon energies (e.g., 2473.0 and 2475.5 eV) the spectra increasingly resemble those of pure P3HT. These measurements also reveal resonant enhancement and branching of decay channels, reflecting the energy-dependent interplay between localized and delocalized states at the hybrid interface. To obtain a more quantitative understanding of this behavior, Figure 5 analyzes the individual decay channels of each species, as determined from spectral deconvolution.

**Figure 4:**
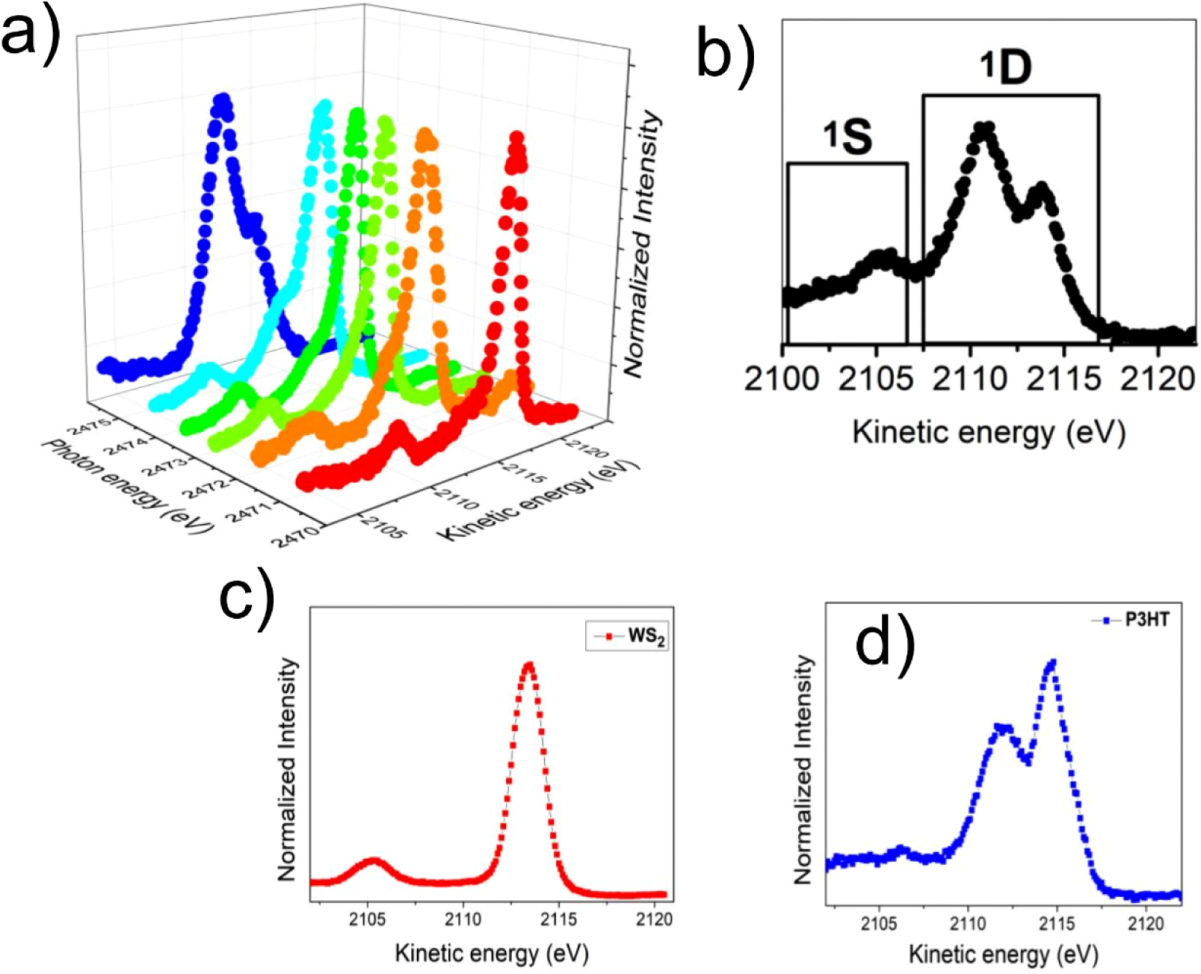
(a) S–KL_2,3_L_2,3_ resonant Auger (RAS) spectra of P3HT–WS_2_/SiO_2_ acquired at different excitation energies across the S K-edge. (b) Nonresonant Auger spectrum of P3HT–WS_2_/SiO_2_ recorded at 2510 eV, showing the ^1^S and ^1^D multiplet components. (c) S–KL_2,3_L_2,3_ RAS spectrum of pristine WS_2_/SiO_2_ measured at 2471.0 eV. (d) S–KL_2,3_L_2,3_ RAS spectrum of the pure P3HT/SiO_2_ polymer film recorded at 2475.5 eV.

**Figure 5:**
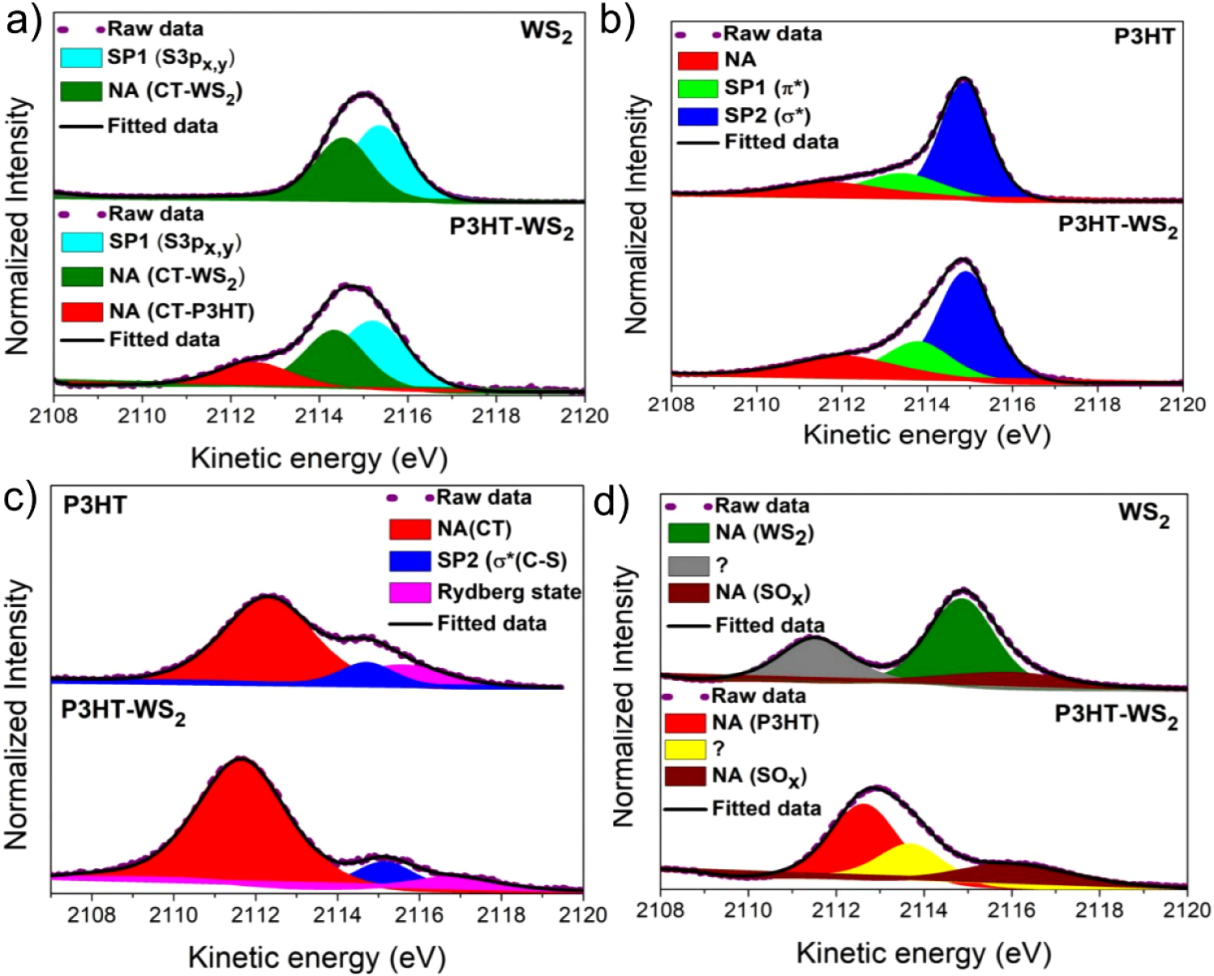
Fitted S–KL_2,3_L_2,3_ RAS spectra of WS_2_/SiO_2_, P3HT/SiO_2_, and P3HT–WS_2_/SiO_2_ films, showing contributions from the Raman spectator decay channels and the normal Auger (CT) channel. Spectra were acquired at photon energies corresponding to (a) the S 1s → 3p_*x*,*y*_ transition (2471.0 eV), (b) the S 1s → π* (S=C) transition (2473.0 eV), (c) the S 1s → σ* (S–C) transition (2475.4 eV), and (d) an excitation energy above the S 1s ionization threshold (2482.0 eV).

Figure 5 presents a comparison of the S–KL_2,3_L_2,3_ Auger spectra of the isolated films (WS_2_/SiO_2_ and P3HT/SiO_2_) and the P3HT–WS_2_ nanocomposite, collected at three different photon energies within the S K-edge resonance. These excitation energies correspond to the S 1s → 3p_*x*,*y*_ transitions (2471.0 eV) of WS_2_, as well as the S 1s → π* (2473.0 eV) and S 1s → σ*­(2475.4 eV) transitions of the P3HT polymer. The RAS spectra of P3HT were deconvoluted into two spectator Auger decay channels: the π* (S=C) (SP1) and σ* (S–C) (SP2) features, along with a normal Auger contribution appearing at a constant kinetic energy of approximately 2112.0 eV.^38,55,58–60^ The main fitting parameters and their corresponding standard deviations are summarized in Tables SI3–SI5. Additionally, Rydberg states emerge in the RAS spectra collected at photon energies approaching the sulfur ionization threshold.^55^ Similarly, the S–KL_2,3_L_2,3_ Auger spectra of WS_2_ also display spectator features associated with electrons excited to the S 3p_*x*,*y*_ (SP1) and S 3p_*z*_ (SP2) states, along with a normal Auger component that appears at a constant kinetic energy of around 2114.5 eV.^53^ The distinction between spectator and charge transfer (CT) decay channels in the RAS spectra relies on the kinetic energy behavior of the Auger electron as the photon energy varies across the resonance. Spectator decays show a photon-dependent shift in kinetic energy due to screening effects, while CT decays yield a constant kinetic energy, characteristic of normal Auger processes. In addition to this distinction, another established criterion is that the intensity variation of spectator channels follows the same photon-energy dependence as the corresponding NEXAFS spectrum. This correlation is illustrated in Figure SI1, where the intensity of the SP2 spectator feature in P3HT closely reproduces the NEXAFS profile, further corroborating the assignment of this decay channel. Such behavior in WS_2_ and P3HT films has also been consistently reported in earlier studies by our group.^37,38,53,55^ Additionally, the Auger spectra, recorded at a photon energy of 2482.0 eV, i.e., above the S 1s ionization threshold, for WS_2_ and P3HT–WS_2_ samples are also shown in Figure 5d. The observed differences in the nonresonant S–KL_2,3_L_2,3_ Auger spectra at 2482.0 eV between WS_2_ and P3HT–WS_2_ can be traced to sulfur oxidation in the composite. The formation of sulfate-like SO_*x*_ species modifies final-state screening and core-hole relaxation, leading to shifts and changes in line shapes of the S–KL_2,3_L_2,3_ decay, a behavior consistent with earlier studies on chemical-state–dependent Auger shifts in sulfur compounds.^61–63^ In particular, oxidized sulfur species (SO_*x*_) typically give rise to S–KL_2,3_L_2,3_ decay Auger peaks at higher kinetic energies compared to sulfide states, with reported shifts of approximately +4–6 eV. Some of the remaining features in the spectra could not be unambiguously assigned, as their identification would require further theoretical calculations or detailed studies of oxidized sulfur species, which are beyond the scope of the present work.

To quantify the ultrafast charge transfer dynamics in these systems, we applied the core-hole clock (CHC) approach, which enables femtosecond-scale estimation of electron delocalization times based on resonant Auger decay behavior. The working principle of the CHC method is illustrated in Scheme 1. In this process, X-ray photon absorption promotes a core-level electron to an unoccupied state, creating a transient core hole (Scheme 1a). The subsequent decay channels depend on whether the excited electron remains localized or delocalizes within the core-hole lifetime. If the electron remains localized, two Raman-like processes can occur: (i) a spectator decay (Scheme 1B), where the excited electron does not participate in the decay, resulting in a final state with two holes and one electron (2h1e); and (ii) a participator decay (Scheme 1C), in which the excited electron either fills the core hole or is emitted, yielding a single-hole (1h) final state. Conversely, if the excited electron delocalizes before core-hole decay (Scheme 1D), a charge transfer (CT) occurs, leading to a two-hole (2h) final state, akin to conventional Auger decay. By comparing the relative intensities of the localized (Raman) and delocalized (CT-Auger) decay channels assumed to act independently the characteristic charge transfer time (τ_CT_) can be extracted. This time scale is referenced against the intrinsic core-hole lifetime (τ_CH_), which serves as an internal clock. Accurate τ_CT_ values are typically accessible when they fall within 0.1 to 10 times τ_CH_, depending on the spectral resolution and signal quality.^27^

**Scheme 1:**
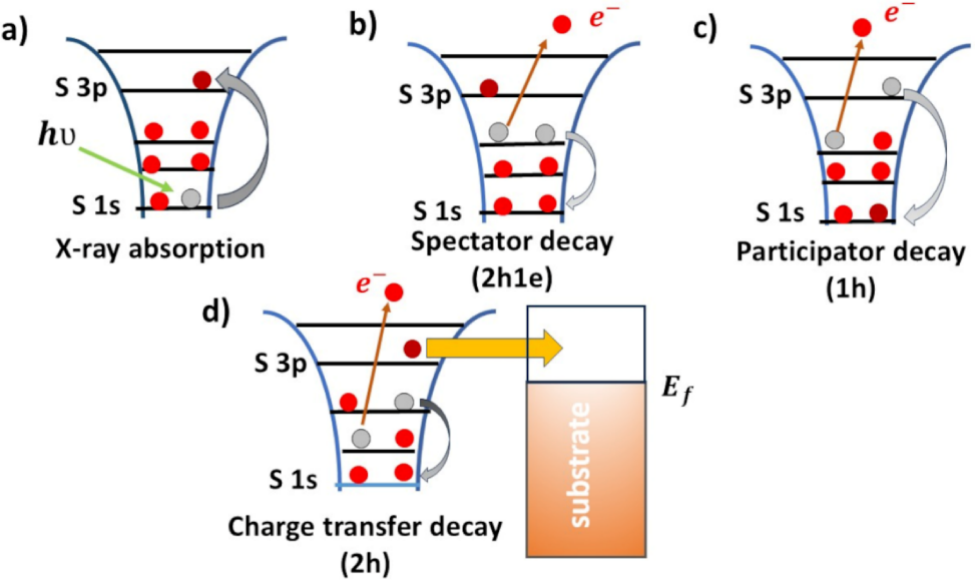
Conceptual representation of the core-hole clock technique^a^

a (a) A core-level electron is resonantly excited into an unoccupied electronic state, generating a core hole. (b) In the spectator decay, the excited electron remains localized and does not partake in the decay, resulting in a final state with two holes and one electron (2h1e). (c) In the participator decay, the excited electron directly contributes to the decay, either by refilling the core hole or being emitted, yielding a single-hole (1h) final state. (d) In the charge transfer (CT) scenario, the excited electron delocalizes to the surrounding matrix before decay, giving rise to a standard Auger-like process and a two-hole (2h) final state.

The charge transfer times (τ_CT_) for the three samples were calculated from the ratio of spectator to normal Auger signal intensities using the equation τ_CT_ = (*I*_spectator_/*I*_normal_) × τ_CH_,^27^ with the S 1s core-hole lifetime (τ_CH_ = 1.27 fs) serving as the internal clock.^64^ To estimate the τ_CT_ in the P3HT–WS_2_ composite, only the WS_2_-derived decay channel was considered, using data acquired at the excitation energy of 2471 eV. No significant change was observed in the τ_CT_ values estimated for the S 1s → 3p_*x*,*y*_ transition at 2471.0 eV between pristine WS_2_ (1.62 ± 0.6 fs) and the P3HT–WS_2_/SiO_2_ nanocomposite (1.58 ± 0.5 fs). The ultrafast charge transfer from this excited state is attributed to the strong hybridization between W 5d and S 3p orbitals in the covalent W–S bonds.^53^ However, for electrons excited into the π* (S=C) and σ* (S–C) molecular orbitals of the P3HT polymer, the charge transfer times were noticeably influenced by electronic interactions with WS_2_. Specifically, for excitation into the π* (S=C) orbital at 2473.0 eV, τ_CT_ decreased from 8.3 ± 0.5 fs in pure P3HT polymer film to 4.8 ± 0.5 fs in the nanocomposite. A similar trend was observed for excitation into the σ* (S–C) orbital at 2475.4 eV, where τ_CT_ dropped from 2.71 ± 0.5 fs in pure P3HT polymer film to 1.05 ± 0.5 fs in P3HT–WS_2_/SiO_2_. The enhancement in charge transfer efficiency upon incorporation of WS_2_ can be attributed to the establishment of strong interfacial electronic coupling between the donor (P3HT) and acceptor (WS_2_) components. This coupling likely facilitates ultrafast electron delocalization across the interface, as evidenced by the shorter τ_CT_ values observed for P3HT states in the nanocomposite. In addition, the presence of WS_2_ may introduce new electronic states^65^ or hybridized orbitals that serve as efficient charge-transfer channels,^63^ further promoting faster electron extraction from the polymer domains. Although NEXAFS results indicate that the incorporation of WS_2_ nanosheets disrupts the molecular ordering of P3HT, they simultaneously point to an improvement in charge transfer dynamics. This behavior parallels that observed in P3HT/PCBM blends, where the formation of polymer–acceptor domains similarly reduce polymer crystallinity while enhancing interfacial charge separation. For example, Liu et al.^66^ reported that morphological disorder at the P3HT/PCBM interface still supports fast charge-transfer rates, and Dimitriev et al.^59^ demonstrated that chain disorder facilitates exciton dissociation and promotes more efficient polaron formation, despite the loss of molecular order. Conversely, Johansson et al.^67^ investigated charge transfer dynamics at a donor–acceptor interface using the core-hole clock approach for the thiophene-based polymer PCPDTBT ([poly­[2,6-(4,4-bis­(2-ethylhexyl)-4H-cyclopenta­[2,1-b;3,4-b′]­dithiophene)-*alt*-4,7-(2,1,3-benzothiadiazole)]]) blended with the fullerene derivative PCBM ([6,6]-phenyl-C_61_-butyric acid methyl ester). They found that a PCPDTBT/PCBM blend with a 1:2 weight ratio exhibited an 86% reduction in charge-transfer time, attributed to conformational ordering of the polymer chains that alters their self-organization and orbital overlap, thereby enhancing interfacial charge transfer. These observations are consistent with our findings, where structural reorganization similarly plays a central role in facilitating ultrafast charge transfer at the hybrid interface.

To gain further insight into the charge transfer mechanism in the nanocomposite, we analyzed the excitation-energy dependence of the charge transfer times, as shown in Figure 6. This figure presents the charge transfer times (τ_CT_) for the P3HT/SiO_2_ and P3HT–WS_2_/SiO_2_ samples as a function of excitation energy. Both systems exhibit an approximately exponential decrease in τ_CT_ with increasing excitation energy, which is characteristic of a tunneling-mediated electron transfer mechanism.^68^ However, the exponential fits reveal distinct slopes for each case. The gradual slope observed for the P3HT–WS_2_/SiO_2_ nanocomposite, relative to the pure P3HT polymer film, suggests broader bandwidths of delocalized states in the nanocomposite, consistent with enhanced electronic coupling between WS_2_ and P3HT. Similar exponential decay behavior was reported by Berggren et al.,^69^ who investigated charge transfer in blends (10, 50, 90, and 100% by weight) of the p-type polymer P­(g42T-T) (bithiophene–thiophene) and the n-type polymer BBL (poly­(benzimidazo-benzo-phenanthroline)) using sulfur KL_2,3_L_2,3_ resonant Auger spectroscopy. In that study, the 50% blend exhibited the most efficient interpolymer charge transfer, while blends containing 10% or 90% acceptor were less efficient, and the 0% blend showed the lowest efficiency. These results demonstrate that interpolymer charge transfer at the donor–acceptor interface is more efficient than intrapolymer pathways, fully consistent with our observations that incorporation of WS_2_ enhances charge transfer in P3HT through effective donor–acceptor interactions.

**Figure 6:**
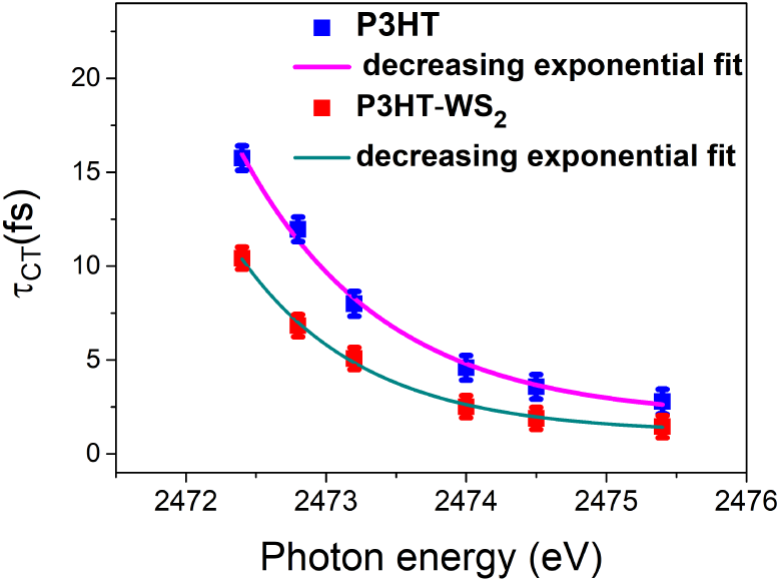
Charge transfer time (τ_CT_) as a function of photon energy for pristine P3HT and the P3HT–WS_2_ nanocomposite. The data are fitted with a decreasing exponential function. Error bars represent a standard deviation of 3% for each data point. The pink and dark cyan curves correspond to the exponential fits for P3HT and P3HT–WS_2_, respectively.

## 4 Conclusion

In summary, we have investigated the ultrafast charge transfer dynamics in P3HT–WS_2_ nanocomposites by combining core-hole clock spectroscopy with AFM and sulfur K-edge NEXAFS analysis. AFM measurements revealed that the incorporation of WS_2_ nanosheets leads to the formation of distinct domains within the polymer matrix, indicating a significant modification of its nanoscale morphology. Valence band XPS analysis showed a shift in the HOMO onset from 1.52 eV in pure P3HT polymer film to 0.98 eV in the nanocomposite, supporting the formation of a donor–acceptor interface and indicating interfacial charge transfer from P3HT to WS_2_. Sulfur K-edge NEXAFS spectra of the composite displayed characteristic features of both P3HT and WS_2_, along with an enhanced postedge resonance, suggesting the presence of electronic hybridization between the two components. Angular-dependent NEXAFS analysis further revealed that WS_2_ incorporation disrupts the preferential molecular orientation of P3HT chains, leading to reduced structural order in the polymer. CHC spectroscopy enabled direct quantification of charge transfer times from specific electronic states. For electrons excited into the WS_2_ S 3p_*x* *y*_ orbitals, no significant variations in charge transfer times were observed between pristine WS_2_ and the P3HT-WS_2_ nanocomposite, suggesting limited interfacial coupling from these states. In contrast, electrons excited into the π* (C=S) and σ* (C–S) molecular orbitals of P3HT exhibited significantly faster charge transfer in the nanocomposite, indicating enhanced interfacial electronic coupling mediated by the WS_2_ nanosheets. The excitation energy-dependence of charge transfer times revealed a characteristic exponential decay, consistent with a tunneling-mediated electron transfer mechanism operating in both systems. These findings highlight the potential of TMDs–polymer nanocomposites to tailor interfacial dynamics on femtosecond time scales, offering a promising strategy for the rational design of next-generation optoelectronic and energy-conversion devices.

## Supplementary Material



## Notes

Table SI: main fitting parameters of the S 2p XPS spectra for pure P3HT polymer and P3HT–WS_2_ films; Table SI2: main fitting parameters of the W 4f XPS spectra for pristine WS_2_ and P3HT–WS_2_ films; Table SI3: main parameters obtained from the fitting of sulfur-KL_2,3_L_2,3_ RAS spectra of P3HT thin film collected at various X-ray photon energies using the sum (SGL) form Pseudo-Voigt profile functions; Table SI4: main parameters obtained from the fitting of sulfur Sulfur-KL_2,3_L_2,3_ RAS spectra of WS_2_/SiO_2_ film collected at various X-ray photon energies using the sum (SGL) form Pseudo-Voigt profile functions; Table SI5: main parameters obtained from the fitting of sulfur sulfur-KL_2,3_L_2,3_ RAS spectra of P3HT-WS_2_ film collected at various X-ray photon energies using the sum form (SGL) form Pseudo-Voigt profile functions; Figure SI1: photon-energy dependence of the SP2 spectator intensity in P3HT compared with the normalized NEXAFS spectrum; the similarity between the two profiles demonstrates that the spectator channel follows the same photon-energy dependence as the NEXAFS, supporting its assignment (PDF)

## ⊥

School of Physics and Astronomy, University of Manchester, Manchester M13 9PL, U.K
