# Supplementary material for: Probing Femtosecond Charge Transfer Dynamics in P3HT–WS2 Nanocomposites via Resonant Core Hole Clock Spectroscopy
Source: ACS Omega. 2025 Oct 13;10(41):48750–60. doi: 10.1021/acsomega.5c06764 (PMC12547753; doi:10.1021/acsomega.5c06764)
Supplement: Supplementary file 1 [file ao5c06764_si_001.pdf]

Supplementary information file

Probing Femtosecond Charge Transfer Dynamics in P3HT–WS_2 Nanocomposites via Resonant Core Hole Clock Spectroscopy.

Yunier Garcia-Basabe,[§,†,] * Matheus Suenson Cardoso, [‡, ⊥,†] Jorge Arce-Molina, [‡, †] and Dunieskys G. Larrude [‡, †].

[,§,] *Universidade Federal da Integração Latino-Americana, UNILA, 85867-970, Foz do Iguaçu, Brazil.*

[‡] *School of Engineering, Mackenzie Presbyterian University, São Paulo 01302-907, Brazil.*

** Corresponding author:*
Email: yunier.basabe@unila.edu.br
yunierbasabe26@gmail.com
tel: +55-453529 2113

[⊥] Present Address: School of Physics and Astronomy, University of Manchester, Manchester M13 9PL, U.K.

[†] These authors contributed equally to this work.

Contents:

- **Table SI**. Main fitting parameters of the S 2p XPS spectra for pure P3HT polymer and P3HT–WS_2 films.
- **Table SI2**. Main fitting parameters of the W 4f XPS spectra for pristine WS_2 and P3HT–WS_2 films.
- **Table SI3**. Main parameters obtained from the fitting of sulfur Sulfur-$KL_{2,3}L_{2,3}$ RAS spectra of P3HT thin film collected at various X-ray photon energies using the sum form (SGL) form Pseudo-Voigt profile functions.
- **Table SI4**. Main parameters obtained from the fitting of sulfur Sulfur-$KL_{2,3}L_{2,3}$ RAS spectra of WS_2/SiO_2 film collected at various X-ray photon energies using the sum form (SGL) form Pseudo-Voigt profile functions.
- **Table SI5**. Main parameters obtained from the fitting of sulfur Sulfur-$KL_{2,3}L_{2,3}$ RAS spectra of P3HT-WS_2 film collected at various X-ray photon energies using the sum form (SGL) form Pseudo-Voigt profile functions.
- **Figure SI1**. Photon-energy dependence of the SP2 spectator intensity in P3HT compared with the normalized NEXAFS spectrum. The similarity between the two profiles demonstrates that the spectator channel follows the same photon-energy dependence as the NEXAFS, supporting its assignment.

Table SI1. Main fitting parameters of the S 2p XPS spectra for pure P3HT polymer and P3HT–WS_2 films.

Samples	$S\ 2p_{3/2}$			$S\ 2p_{1/2}$			SO_4^{2-}		
	**BE (eV)**	**FWHM (eV)**	**At %**	**BE (eV)**	**FWHM (eV)**	**At. %**	**BE (eV)**	**FWHM (eV)**	**At. %**
**P3HT**	164.2	1.64	66.7	165.4	1.64	33.3	-	-	-
**P3HT-WS_2**	164.3	1.13	35.1	165.5	1.13	17.5	169.5	1.68	25.4

Table SI2. Main fitting parameters of the W 4f XPS spectra for pristine WS_2 and P3HT–WS_2 films.

**Samples**	**W 4f$_{7/2}$**			**W 4f$_{5/2}$**			**WO_3**		
	**BE (eV)**	**FWHM (eV)**	**At %**	**BE (eV)**	**FWHM (eV)**	**At. %**	**BE (eV)**	**FWHM (eV)**	**At. %**
**WS_2**	33.7	1.29	66.7	35.8	1.29	33.3	36.9	1.55	16.1
**P3HT-WS_2**	33.6	1.10	35.1	35.7	1.10	17.5	36.8	1.33	21.7

Table SI3. Main fitting parameters of the sulfur $KL_{2,3}L_{2,3}$ RAS spectra of the P3HT thin film collected at various X-ray photon energies, obtained using the sum (SGL) Pseudo-Voigt profile functions. Values in parentheses correspond to the standard deviations of the fitted parameters.

**hν (eV)**	**2473.0**			**2474.5**		
	**KE (eV)**	**FWHM (eV)**	**A (%)**	**KE (eV)**	**FWHM (eV)**	**A (%)**
**SP1 (π*)**	**2113.6 (5)**	**1.78 (4)**	**11.0 (6)**	**-**	**-**	**-**
**SP2 (σ *)**	**2114.9 (3)**	**1.31(5)**	**60.0 (5)**	**2115.7 (4)**	**2.18 (5)**	**25.8 (9)**
**NA**	**2112.2 (3)**	**3.14 (6)**	**10.9 (5)**	**2111.9 (5)**	**1.37 (6)**	**51.2 (5)**
**Rydberg states**	**-**	**-**	**-**	**2117.4 (4)**	**1.62 (5)**	**23.6 (8)**

Table SI4. Main fitting parameters of the sulfur $KL_{2,3}L_{2,3}$ RAS spectra of the WS_2/SiO_2 film collected at various X-ray photon energies, obtained using the sum (SGL) Pseudo-Voigt profile functions. Values in parentheses correspond to the standard deviations of the fitted parameters.

**hv (eV)**	**2471.0**			**2473.0**		
	**KE (eV)**	**FWHM (eV)**	**A (%)**	**KE (eV)**	**FWHM (eV)**	**A (%)**
**SP1 (S $3p_{x,y}$)**	**2115.2 (5)**	**1.49 (4)**	**50.1 (6)**	**2116.7 (5)**	**1.61 (5)**	**12.5 (5)**
**SP2 (S $3p_z$)**	**-**	**-**	**-**	**2118.3 (4)**	**1.63 (6)**	**9.6 (7)**
**NA**	**2114.5 (6)**	**1.54 (4)**	**38.7 (5)**	**2114.6 (5)**	**1.50 (7)**	**65.2 (9)**

Table SI5. Main fitting parameters of the sulfur $KL_{2,3}L_{2,3}$ RAS spectra of the P3HT–WS_2 film collected at various X-ray photon energies, obtained using the sum (SGL) Pseudo-Voigt profile functions. Values in parentheses correspond to the standard deviations of the fitted parameters.

**hv (eV)**	**2471.0**			**2473.0**			**2475.4**		
	**KE (eV)**	**FWHM (eV)**	**at (%)**	**KE (eV)**	**FWHM (eV)**	**at (%)**	**KE (eV)**	**FWHM (eV)**	**at (%)**
**SP1 (S $3p_{x,y}$)**	**2116.3 (4)**	**-**	**31.4 (5)**	**-**	**-**	**-**	**-**	**-**	**-**
**π* (S=C)**	**-**	**-**	**-**	**2113.9 (5)**	**1.73 (6)**	**16.5 (5)**	**-**	**-**	**-**
**σ *(S-C)**	**-**		**-**	**2115.1 (4)**	**1.50 (4)**	**58.5 (5)**	**2115.8 (3)**	**1.96 (3)**	**29.5 (7)**
**NA (CT-WS_2)**	**2114.2 (4)**	**1.91(3)**	**55.0 (4)**	**-**	**-**	**-**		**-**	
**NA (CT-P3HT)**	**2112.0 (5)**	**2.57(6)**	**12.0 (5)**	**2111.9 (3)**	**2.69 (7)**	**19.1 (5)**	**2112.0 (1)**	**2.5 (4)**	**50.5 (3)**
**Rydeberg states**	**-**	**-**	**-**	**-**	**-**	**-**	**2117.0 (3)**	**2.4 (4)**	**11.2 (4)**

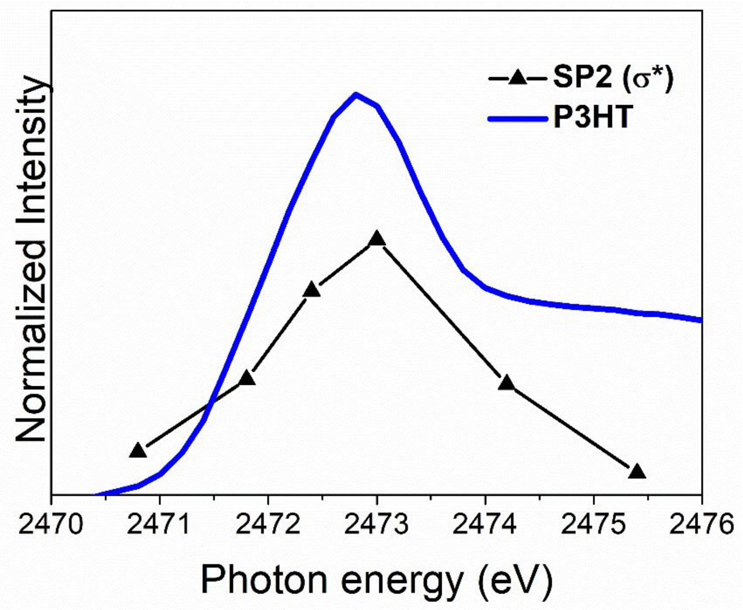

Figure SI1. Photon-energy dependence of the SP2 spectator intensity in P3HT compared with the normalized NEXAFS spectrum. The similarity between the two profiles demonstrates that the spectator channel follows the same photon-energy dependence as the NEXAFS, supporting its assignment.
